# The Function and related Diseases of Protein Crotonylation

**DOI:** 10.7150/ijbs.58872

**Published:** 2021-08-09

**Authors:** Shuo Wang, Guanqun Mu, Bingquan Qiu, Meng Wang, Zunbo Yu, Weibin Wang, Jiadong Wang, Yang Yang

**Affiliations:** 1 Department of Biochemistry and Molecular Biology, Beijing Key Laboratory of Protein Posttranslational Modifications and Cell Function, School of Basic Medical Sciences, School of Basic Medical Sciences, Peking University Health Science Center, Beijing 100191, China.; 2 China Institute of Veterinary Drugs Control, Beijing 100181, China.; 3 Department of Radiation Medicine, Institute of Systems Biomedicine, School of Basic Medical Sciences, School of Basic Medical Sciences, Peking University Health Science Center, Beijing 100191, China.

**Keywords:** crotonylation, histone crotonylation, non-histone crotonylation, disease

## Abstract

Crotonylation is a kind of newly discovered acylation modification. Thousands of crotonylation sites have been identified in histone and non-histone proteins over the past decade. As a modification closely related to acetylation, crotonylation was reported to share many universal enzymes with acetylation. Crotonylated proteins have important roles in the regulation of various biological processes, such as gene expression, process of spermatogenesis, cell cycle, and also in the pathogenesis of different diseases, which range from depression to cancer. In this review, we summarize the research processes of crotonylation and discuss the advances of regulation mechanism of both histone and non-histone proteins crotonylation in difference physiological processes. Also, we focus on the alteration of the crotonylation under certain pathological conditions and its role in the pathogenesis of each disease.

## Introduction

Scientists have focused on epigenetic mechanism study since the concept of Epigenetics was firstly proposed by Austrian developmental biologist Conrad Waddington in 1942 1. In addition to DNA methylation, histone modifications have been found as a main component of the epigenetic code. Over the last ten years, a variety of histone acylation modifications have been discovered, including histone propionylation (Kpr) 2, butyrylation (Kbu) 2, 2-hydroxyisobutylacylation (Khib) 3, succinylation (Ksucc) 4, malonylation (Kma) 4, glutarylation (Kglu) 5, crotonylation (Kcr) 6, β-hydroxybutyrylation (Kbhb) 7, and benzoylation (Kbz) 8. These modifications arise from their corresponding acyl-CoAs. Familiar with lysine acetylation, acylation mainly occurs on lysine residues through the addition of an acyl group from an acyl-CoA donor to the ε-amino group of the lysine side chain. However, there still have distinctions in chemical properties between different acylation. Kpr, Kbu, Kcr and Kbz belong to hydrophobic groups which can neutralise the positive charge of lysine residues (like acetylation) 9. KSucc, Kmal and Kglu belong to acidic groups which change the positive charge to a negative charge. Khib and Kbhb belong to polar groups which allow hydrogen bond formation with interacting molecules 9. Kbz stands out as the only known histone PTM with an aromatic acyl group, while Kcr is planar and Kbhb and Khib are branched 9. The effect of structural biochemistry of acylation needs to further explore. Some data have shown that the known HAT and HDAC families have wide-ranging acylation and deacylation capabilities. Also, some acylation modifications were also found in non-histone proteins, indicating that protein acylation modifications are widely distributed in cells and play important roles in physiological and pathological processes.

Lysine crotonylation (Kcr) is one of histone lysine acylation modifications 6. Crotonylation mainly occurs on the ε-amino group of lysine in histones and was recently reported to occurs on serine residue 10. As mass spectrometry and proteomics developed, the existence of crotonylation has been confirmed in various microorganism (bacteria and fungus), plants and animals 11-13. For example, Zhu J et al. found that Brucella secreted protein F (BspF) owned both acetylase and decrotonylase activity *in Brucella*. The data of LC-MS shown that a total of 5,559 crotonylation sites were identified on 1,525 different proteins, of which 331 sites on 265 proteins were significantly changed in BspF overexpression cells. So the authors speculated that BspF might influence the function of host proteins through its crotonylation to promote the intracellular propagation of *Brucella* 14. This revealed a change of protein crotonylation in host cell mediated by microorganisms and provided a new perspective for further exploring the mechanism of microbial infection. In addition to the universality of crotonylation in various organisms, researches also indicated that crotonylation is a common protein modification which is not only limited to histone proteins, but also has potential effects on non-histone proteins. Histone Kcr chiefly enriches in the enhancer or transcription start site (TSS) of genes in human somatic genome and mouse male germ cell genome, which may activate specific genes 15. What's more, crotonylation has been disclosed to involve in the physiological processes, such as RNA processing, nucleotide metabolism, chromatin recombination, regulation of protein activity and protein localization. Furthermore, crotonylation has countless ties with numerous diseases, like acute kidney injury (AKI) 16, IgA nephropathy 17, depression 18, HIV latency 19 and cancer 20. This review summarizes the recent studies of crotonylation in detail and focuses on the mechanism and function of crotonylation both in histone and non-histone proteins in various physiological processes and different diseases.

### The Research Progress of Crotonylation

**Figure 1:**
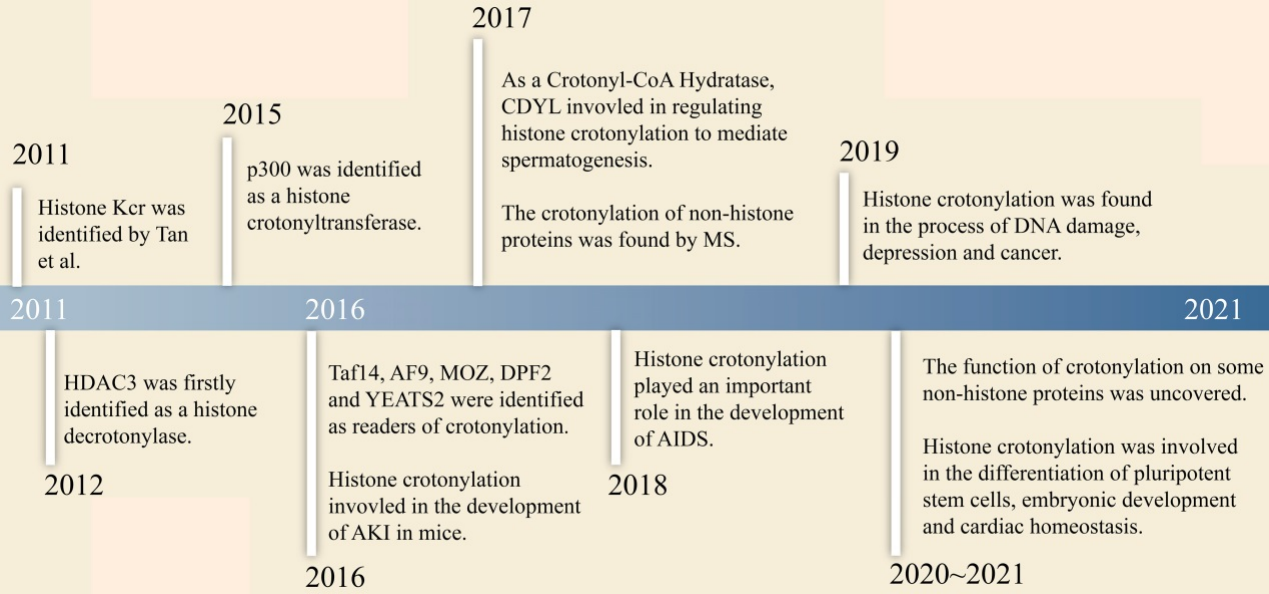
The research history of crotonylation.

The research history of crotonylation is shown in Figure 1. In 2011, Tan et al. firstly reported that histone Kcr labelled either active promoters or potential enhancers in both human somatic cell and male mouse germ cell genomes 6. Further research found that histone Kcr marked certain specific X-linked genes that escaped inactivation of sex chromosomes in haploid cells right after meiosis 6. This study revealed a possible relationship between crotonylation and gene activation. Furthermore, Montellier et al. reported that histone Kcr was a dominant element in maintaining some genes activation in the globally repressive environment of haploid cell sex chromosomes by conferring resistance to transcriptional repressors 15. This indicated that histone Kcr might be an indicator of the male haploid cell gene expression program. Subsequently, Sin et al. disclosed that RNF8 (an E3 ubiquitin-protein ligase) induced multiple modifications including Kcr, which led to gene activation from inactive sex chromosomes in post-meiotic spermatids. Kcr accumulated at transcriptional start sites of sex-linked genes in an RNF8-dependent manner, and increased the expression of RNF8 related genes 21. Since then, the mechanism of histone Kcr during meiosis and post-meiosis in male germ cells was set forth. In 2017, histone crotonylation was found to play a key role in regulating spermatogenesis. Liu et al. showed that chromodomain Y-like transcription corepressor (CDYL) acted as a crotonyl-CoA hydratase and downregulated histone crotonylation. The level of histone crotonylation in round spermatids cells was significantly higher than those in spermatocytes cells, indicating that CDYL negatively regulated histone lysine crotonylation in spermatogenesis 22.

### The Writers/ Crotonyltransferases of crotonylation

In 2015, Sabari et al. showed that the coactivator p300 had both acetyltransferase and crotonyltransferase activities and found that p300-catalyzed histone crotonylation directly stimulated transcription to regulate gene expression 23. Thus, a dynamic relationship between crotonylation and acetylation was speculated. Another study revealed that histone acetyltransferase (HAT) KAT8 (MOF) owns histone crotonyltransferase (HCT) activity, catalyzing the crotonylation in histone H3K4, H3K9, H3K18, H3K23, and histone H4K8 and H4K12 24. Also, CBP and p300 were found to be the major HCT in mammalian cells. CBP/p300 mutants with deficient HAT but competent HCT activity were able to substitute the endogenous CBP/p300 to enhance transcriptional activation, which correlated with enhanced promoter crotonylation and the recruitment of histone crotonylation reader protein Zinc finger protein ubi-d4 (DPF2) 24. Another HAT, KAT2B (PCAF), was also confirmed as a HCT 25. Also, Kollenstart et al. identified that HAT complex Gcn5-Ada2-Ada3 (ADA) and Esa1-Yng2-Epl1 (Piccolo NuA4) crotonylated histones in the N-terminal tails of histone H3 and H4 in budding yeast to promote crotonylation-dependent transcription 26.

### The Erasers/Decrotonylases of crotonylation

The decrotonylases remove the covalent modification of lysine crotonylation. In 2012, histone deacetylase 3 (HDAC3) was firstly reported to own the activity of histone decrotonylase (HDCR) 27. By systematic screening of the activities of the eleven human zinc-dependent lysine deacylases, HDAC3 in complex with nuclear receptor corepressor 1 (HDAC3-NCoR1) was shown to harbor decrotonylase activity *in vitro* 27. Afterwards, SIRT1, SIRT2 and SIRT3 were indicated to act as histone decrotonylases *in vitro* 28, 29. Wei et al. claimed that HDACs, rather than SIRTs, exerted the main activity of HDCR 30. Histone crotonylation is a dynamic process as histone acetylation in mammalian cells and both catalytic centers of HDAC Class Ι and SIRTs have activities of HDAC and HDCR 30. Kelly et al. found HDAC1/CoREST1/LSD1 complex was involved in both decrotonylation and deacetylation. In embryonic stem cells, knockdown HDAC1/2 increased global crotonylation levels of histone and caused a great reduction in total decrotonylase activity 31. This study deepened the investigation of decrotonylase and further explored the mechanisms of decrotonylase.

### The Readers of crotonylation

Certain specific domains are identified to participate in the process of transcriptional regulation induced by crotonylation. YEATS, Bromodomain and Double PHD finger (DPF) are three classes of domains that recognized acylation. In 2016, Andrews et al. found that transcription initiation factor TFIID subunit 14 (Taf14) was a reader of histone crotonylation 32. Through π-π-π stacking mechanism, the YEATS domain of Taf14 bound to histone H3K9cr 32. Gowans et al. discovered that histone Kcr was dynamically regulated during the yeast cell metabolic cycle (YMC) 33. Under the condition of deficient nutrition, acetylation and crotonylation on the histone H3K9 site was dynamically changed in subsequence of Taf14 recognition, thus suppressing the expression of growth-related genes 33. Several proteins with YEATS domain are identified, including protein AF-9 homolog (Yaf9), YEATS domain-containing protein 1 (protein ENL), protein AF-9 (AF-9), Taf14, and something about silencing protein 5 (Sas5). Li et al. disclosed that AF9 had stronger affinity with Kcr than Kac and regulated gene expression and positively activated transcription in YEATS domain-dependent manner 34. Also, they found that YEATS domain-containing protein 2 (YEATS2) bound to a repertoire of acylated histone peptides with the best preference for histone H3 with crotonylation on lysine 27 (H3K27cr) 35. Except for the YEATS domain, Bromodomain and DPF domain were also involved in the recognition of histone crotonylation. Researchers found that histone acetyltransferase KAT6A (MOZ) and DPF2 with DPF domain performed broad acylation of histone H3K14, including crotonylation (Kcr), butyrylation (Kbu), and propionylation (Kpr) with a preference for H3K14 crotonylation 36. Immunofluorescence and chromatin immunoprecipitation quantitative PCR (ChIP-qPCR) showed that MOZ colocalized with histone H3K14cr in a DPF domain-dependent manner 36.

**Figure 2:**
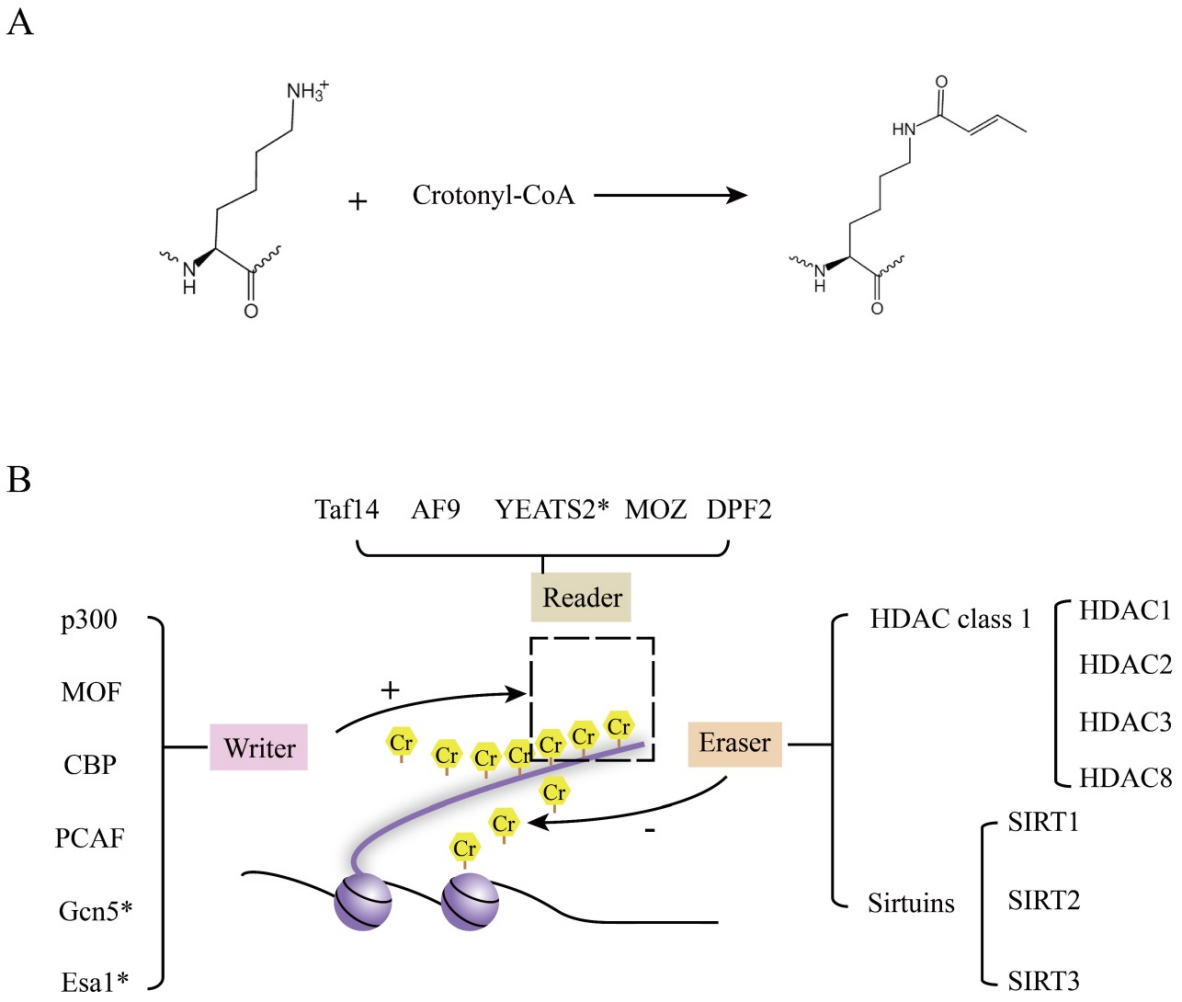
** The chemical formula and regulators of crotonyaltion. A.** The chemical formula and enzymatic reaction of crotonylation 66. **B.** Readers, Writers (crotonyltransferases), and erasers (decrotonylases) are shown in this review. The readers are recruited by protein crotonylation. Writers and Erasers balance the protein crotonylation* in vitro* and* in vivo*. Esa1 and Gcn5 were found in budding yeast. YEATS2 was confirmed* in vitro*. Except for these, the remaining proteins were detected in human.

The chemical formula and the regulators of crotonylation are shown in Figure 2A and 2B. Writers (crotonyltransferases) and erasers (decrotonylases) regulated the dynamic balance of lysine crotonylation, playing different roles in a broad spectrum of cellular processes.

### Other regulation factors of crotonylation

In addition to writers, erasers and readers, there are also other regulatory factors involved in the regulation of crotonylation. As a non-coding RNA, nuclear paraspeckle assembly transcript 1 (NEAT1) was associated with p300/CBP complex and its inhibition affected the location of H3K27 acetylation (H3K27Ac) and H3K27 crotonylation (H3K27Cr) to the transcription start site of many genes, including endocytosis related genes, which promotes the progression of disease 37. Also, acyl-CoA synthetase short-chain family member 2(ASCC2) was a kind of crotonyl-CoA-producing enzyme and it was induced during the infection of HIV. The crotonylation regulated by ASCC2 was involved in the process of HIV reactivation *in vivo* 19. In addition, CDYL was shown to act as a crotonyl-CoA hydratase and downregulated histone crotonylation to play an important role in regulation of crotonylation 22.

## The Function of Crotonylation

### The Function of Histone Crotonylation

**Figure 3:**
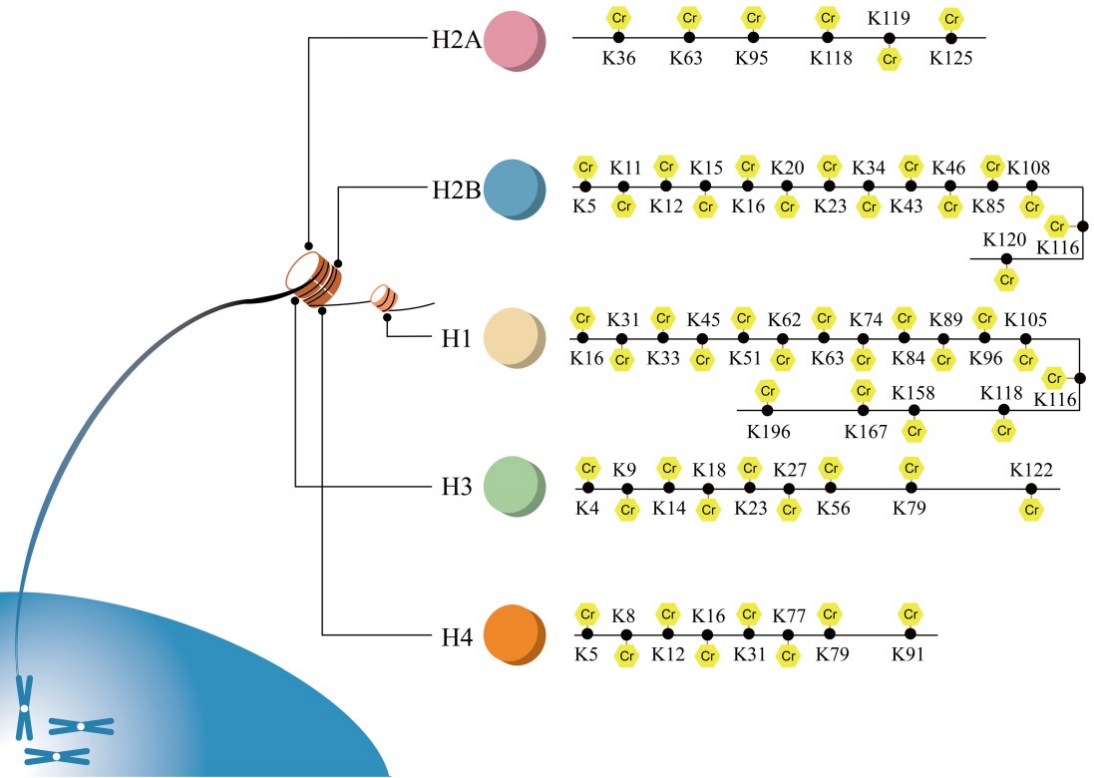
** All sites of histone crotonylation.** Sites of crotonylation were firstly discovered in histone. Until now, all of the sites of histone Kcr of H1, H2A, H2B, H3 and H4 are exhibited.

The identified sites of histone crotonylation are exhibited in Figure 3. Histone crotonylation was firstly reported to regulate gene transcription 6. Histone crotonylation was shown to play roles in gene expression 6, 23, cell cycle 38, spermatogenesis 15, 21, aging 39 and DNA damage 40.

#### Gene expression and transcription

**Figure 4:**
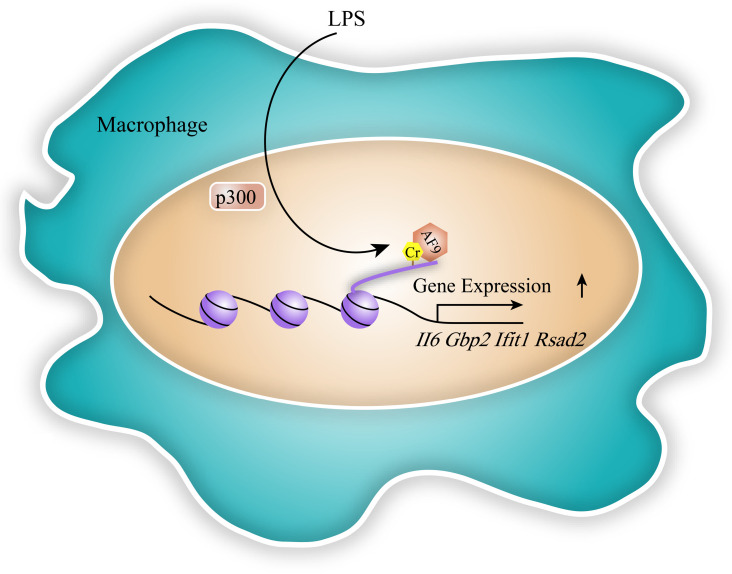
** The function of histone crotonylation in gene expression and transcription.** In the LPS-induced inflammatory response, p300 induces histone crotonylation in TSS and other regulatory elements of target genes such as *Il6*, *Gbp2*, *Ifit1* and *Rsad2*, thereby changing chromosome structure and promoting specific gene expression. AF9, as a reader, recognizes p300-catalyzed crotonylation to participate in the process of gene expression in macrophage.

Transcription is a crucial process in the expression of coding genes 41. Acetylation has been revealed to play a key role in gene expression in past decades. Through locating in TSS and enhancer region of target genes, acetylation relaxed highly condensed chromosomal structure to activate gene expression. Besides histone lysine acetylation, a repertoire of acylation types has been identified to regulate gene expression including histone crotonylation. The role of histone Kcr on gene transcription was firstly revealed by Tan et al. in haploid cells 6. Sabari et al. showed that p300 catalyzed histone crotonylation in TSS and other regulatory elements of target genes such as Interleukin-6 (Il6), Guanylate-binding protein 2 (Gbp2), Interferon-induced protein with tetratricopeptide repeats 1 (Ifit1) and Radical S-adenosyl methionine domain-containing protein 2 (Rsad2) in response to LPS-induced macrophage inflammatory, thereby changing chromosome structure and promoting their expression 23 (Figure 4). Kollenstart et al. disclosed that Gcn5 and Esa1, as histone crotonylases, participated in cell metabolism through regulating gene expression, which was dependent on crotonate dosage 26. During the self-renewal process of ESCs, histone crotonylation mediated by HDAC1 was reported to maintain the expression of transcription factor SOX-2, octamer-binding transcription factor 4 protein (Oct4) and Nanog 30. However, researchers found that certain histone decrotonylation also promoted ESC differentiation by upregulating related gene expression 30. These data suggested that crotonylation may have dual effects on gene expression.

Meanwhile, some readers of histone Kcr were proved to involve in gene expression and transcription. Li et al. discovered that AF9, a reader of histone Kcr, was involved in the recognition of p300-catalyzed crotonylation and gene expression process in the LPS-induced inflammatory response (Figure 4). They showed that AF9 co-localizes with crotonylated histone H3K18 and positively regulates gene expression of *Rsad2*, *II6*, *Ifit1* in a YEATS domain-dependent manner 34. In addition, histone acetylation-binding DPF domains of human MOZ and DPF2 (also known as BAF45d) was shown to be the strongest preference for Kcr. MOZ and H3K14cr colocalized in a DPF-dependent manner, while H3K14cr was enriched in the genes which MOZ targeted in HEK 293T cells 36. Moreover, YEATS2, a subunit of histone acetyltransferase ATAC complex, specifically recognized H3K27cr *in vitro* 35. The significance of histone crotonylation sites involved in gene expression are shown in Table 1.

#### Spermatogenesis

**Figure 5:**
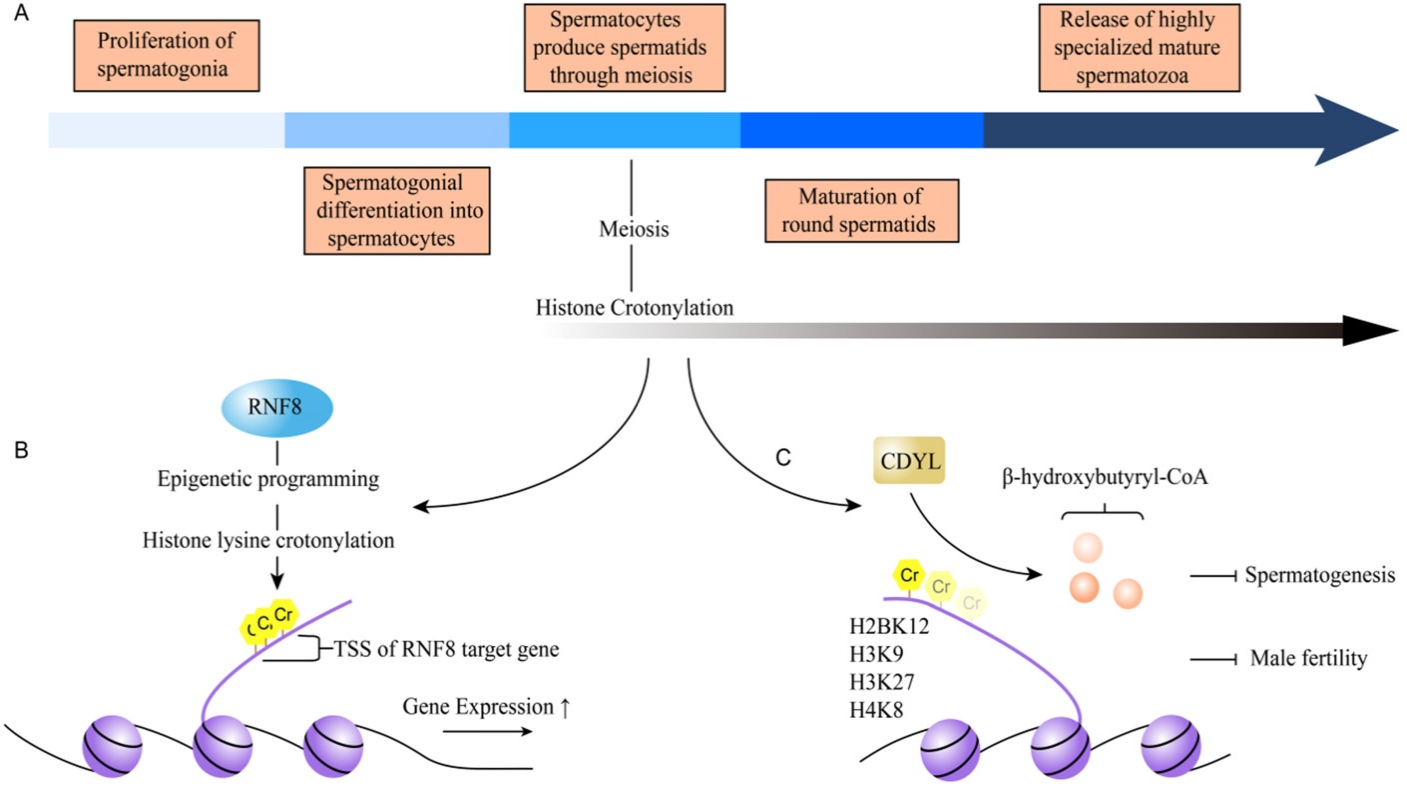
** The mechanism of crotonylation involves in spermatogenesis. A.** The process of spermatogenesis contains the proliferation of spermatogonia, spermatogonial differentiation into spermatocytes, spermatids produced by spermatocytes through meiosis, maturation of round spermatids, and the release of highly specialized mature spermatozoa. **B.** Histone Kcr is one of epigenetic programming caused by RNF-8. It is exceedingly accumulated around TSSs of sex-linked genes in a RNF8-dependent manner and activates gene expression. **C.** CDYL negatively regulates histone crotonylation, to affect spermatogenesis, and the normal function of testicular and male fertility. The main histone crotonylation sites regulated by CDYL are H2BK12, H3K9, H3K27 and H4K8.

Spermatogenesis is a highly conserved physiological process. The complicated process contains five steps: the proliferation of spermatogonia, spermatogonial differentiation into spermatocytes, spermatids produced by spermatocytes through meiosis, maturation of round spermatids, and the release of highly specialized mature spermatozoa 42 (Figure 5A). Numerous factors are identified to affect spermatogenesis, such as obesity, diabetes, environmental chemicals, varicocele and epigenetic factors, especially. DNA content in the male germ cells (GCs) was packed in a very small volume to fit into the sperm head. Specific histones were replaced by protamine via hyperacetylation of histone H4 during spermatid stages, which plays an important role in spermatogenesis 43, 44. Recently, histone Kcr was reported to play an important role in male haploid gene expression and sperm formation after meiosis 15. Histone Kcr does not only mark the sex chromosome-related genes which are active in male germ cells after meiosis, but also mark the activated genes of autosomes after meiosis 15. These studies indicated that histone Kcr may be a dominant factor in maintaining active gene activity in the overall suppressive environment of haploid sex chromosomes due to its resistance to transcription repressor 15. Also, Sin et al. found that RNF8 brought about variation of chromosome conformation in epigenetic programming manner and regulated gene activation from inactive sex chromosomes in post-meiotic spermatids 21. The epigenetic modifications included trimethylation of H3K4, histone Kcr, and incorporation of the histone variant H2AFZ 21. It's worth noting that histone Kcr exceedingly accumulated around TSSs of sex-linked genes in a RNF8-dependent manner and activated gene expression 21 (Figure 5B). The removal of chromatin histones across the genome and their replacement by transition proteins (Tnps) and protamines (Prms) are another unique epigenetic event during spermatogenesis. Liu et al. reported that CDYL acts as a crotonyl-CoA hydratase which converts crotonyl-CoA to β-hydroxybutyryl-CoA and negatively regulates histone crotonylation 22. The main histone crotonylation sites regulated by CDYL were H2BK12, H3K9, H3K27, and H4K8 (Figure 5C). They found that the gene expressions of *peptidyl-prolyl cis-trans isomerase NIMA-interacting 4* (*Pin4*),* coiled-coil domain-containing protein 160* (*Ccdc160*) and *transcription elongation factor A protein-like 1* (*Tceal*) were greatly reduced in RS of CDYL transgenic mice, compared to that in wild-type mice. In addition, ChIP-qPCR assays detected the reduced levels of total histone Kcr and H2BK12cr on the promoter of related genes, showing that CDYL regulated the expression of sex chromosome-linked escaped genes through mainly influencing histone Kcr on the gene promoters to play an important role in spermatogenesis and thus male fertility 22. Together, these results provide us a new insight to understand the role of histone crotonylation in spermatogenesis.

#### Cell cycle

**Figure 6:**
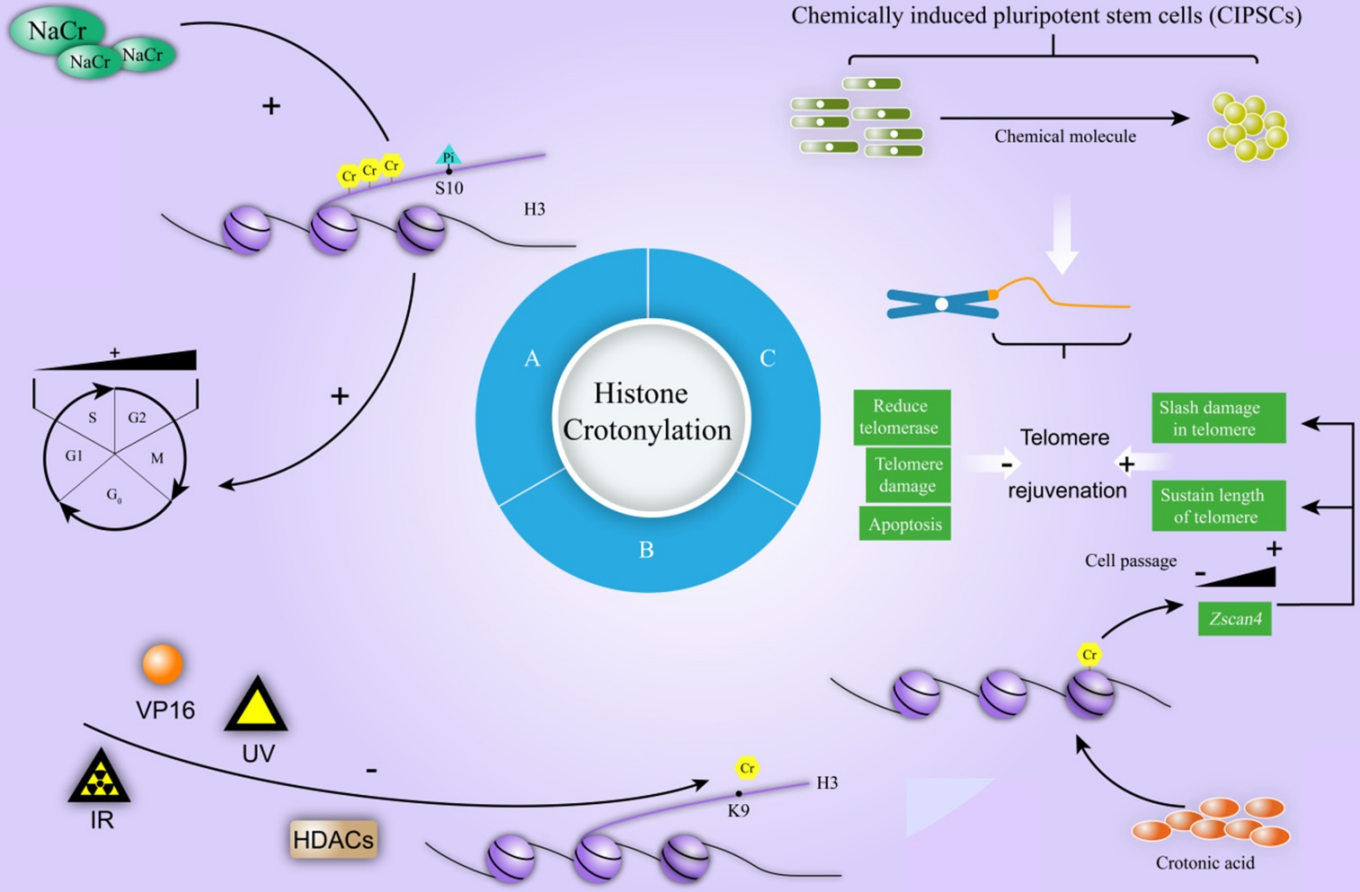
** The mechanism of crotonylation involves in cell cycle, DNA damage repair and CIPSCs. A.** NaCr increases the crotonylation of histone H3 and influences the phosphorylation of histone H3 at the Ser10 site in a dose-dependent manner, which is the mark of G2 / M phase cells. NaCr treatment decreases the amount of S phase cells and raises cells in G2 phase. **B.** Ionizing Radiation (IR), Ultraviolet Radiation (UV), or Etopside (VP16) decreases the levels of H3K9cr. During this process, HDACs are the major HDCR in U2OS cells. **C.** During chemically induced pluripotent stem cells (CIPSCs), telomere rejuvenation goes through a dynamic process. Long-term induction by small molecules reduces telomerase, increases telomere damage and apoptosis, and finally contributes to negatively regulate telomere rejuvenation (shortened telomeres, limited the formation of CIPSCs). Crotonic acid positively regulates telomere rejuvenation through promoting the expression of *Zscan4* with cell passage, which slashes damage in telomere, and sustains the length of telomere during chemically induced reprogramming.

Cell cycle contains four phases (G1, S, G2, and M). Many intracellular and extracellular factors influence this process. During the cell cycle, certain protein has been dynamically regulated by modification, including ubiquitination, methylation, and phosphorylation. Serine phosphorylation, which most appears in the late of G2 stage and surges in H3 in the M stage, has been mostly studied 45. Recently, researchers have initially explored the effect of crotonylation modification during cell cycle. Sodium Crotonate (NaCr) was found to enhance the crotonylation level of histone H3, and influence the phosphorylation of histone H3 at the Ser10 site in a dose-dependent manner, which is the mark of G2 / M phase cells 38. Flow cytometry results further disclosed that the amount of S phase cells declined and G2 phase cells raised after NaCr treatment 38 (Figure 6A). Considering cell cycle comprises four phases and involves complex biological processes, the mechanism of histone crotonylation during cell cycle needs to further investigation.

#### DNA damage response

Post-translational modification plays an important role in DNA damage Response (DDR) 46. Recently, a study shown the role of HDACs in regulating histone crotonylation in DDR 40. After exposure to ionizing radiation (IR), ultraviolet radiation (UV), or treatment with Etopside (VP16), the rapid decrease of H3K9cr levels was detected and the reduction of H3K9cr levels in U2OS cells were transient and could be restored to basal level after few hours 40. The treatment with HDACs inhibitor trichostatin A (TSA) induced severe increase in the levels of H3K9cr, indicating that HDACs are the major lysine decrotonylases (Figure 6B) 40. These data only revealed that H3K9 crotonylation is reduced at damage sites in a HDAC-dependent manner under various DNA damage stimulation and the exact mechanism of histone crotonylation in the process of DNA damage is need to further explore.

#### Telomere

Telomere is a small DNA-protein complex at the ends of the linear chromosomes of eukaryotic cell. Cap-like structure covers the ends of chromosomes to maintain the stability of the genome 47. Telomere length is mainly determined by genes, environmental factors and molecular pathways 48. It is associated with infinite self-renewal and pluripotency, while shortened telomere causes cell senescence or tumorigenesis. Fu et al. disclosed that telomere length strongly correlated with the degree of reprogramming, pluripotency, and differentiation capacity of chemically induced pluripotent stem cells (CIPSCs). Further exploration found that long-term induction by small molecules reduced telomerase, increased telomere damage and apoptosis, and finally contributed to shorten telomeres and limited the formation of CIPSCs 39. Mechanically, crotonic acid induced histone crotonylation activated the expression of *Zinc finger and SCAN domain-containing protein 4 (Zscan4)* gene by decreasing the abundance of heterochromatic H3K9me3 and HP1α at subtelomere. The induced expression of Zscan4 in turn slashed damage in telomere, and sustained length of telomere during chemically induced reprogramming 39 (Figure 6C).

#### Stem Cell Biology

In mouse ES cells, histone decrotonylation was found to promote ES cell differentiation. Histone Kcr was elevated compared to differentiated cells 30. Also, Fu et al. identified the role for histone crotonylation on dynamics of telomere rejuvenation during chemical reprogramming in pluripotent stem cells 39. In 2021, Y. Fang et al reported that histone crotonylation had an important role in the endoderm commitment of human embryonic stem cells 49. They found that H4K77cr and H4K91cr were increased during endoderm differentiation of hESCs 49. In the meanwhile, the expression of key enzymes involved in the metabolism of crotonyl CoA, such as peroxisomal acyl-coenzyme A oxidase 3 (ACOX3), short-chain specific acyl-CoA dehydrogenase (ACADS) and ACSS2, were significantly increased in endoderm differentiation 49. These data indicated that crotonyl-CoA-producing enzymes modulated histone crotonylation and regulated endoderm differentiation. Also, the data of global profiling of the lysine crotonylome in different pluripotent states revealed that the important role of protein crotonylation in the maintenance and transformation of pluripotent stem cells 50. They identified 3628 high-confidence crotonylated sites in 1426 proteins, which are involved in functions/processes related to pluripotency such as RNA biogenesis, central carbon metabolism, and proteasome function 50. These reports indicated the crucial role of histone crotonylation on stem cell biology.

### The Function of non-histone Crotonylation

#### Protein activity

**Figure 7:**
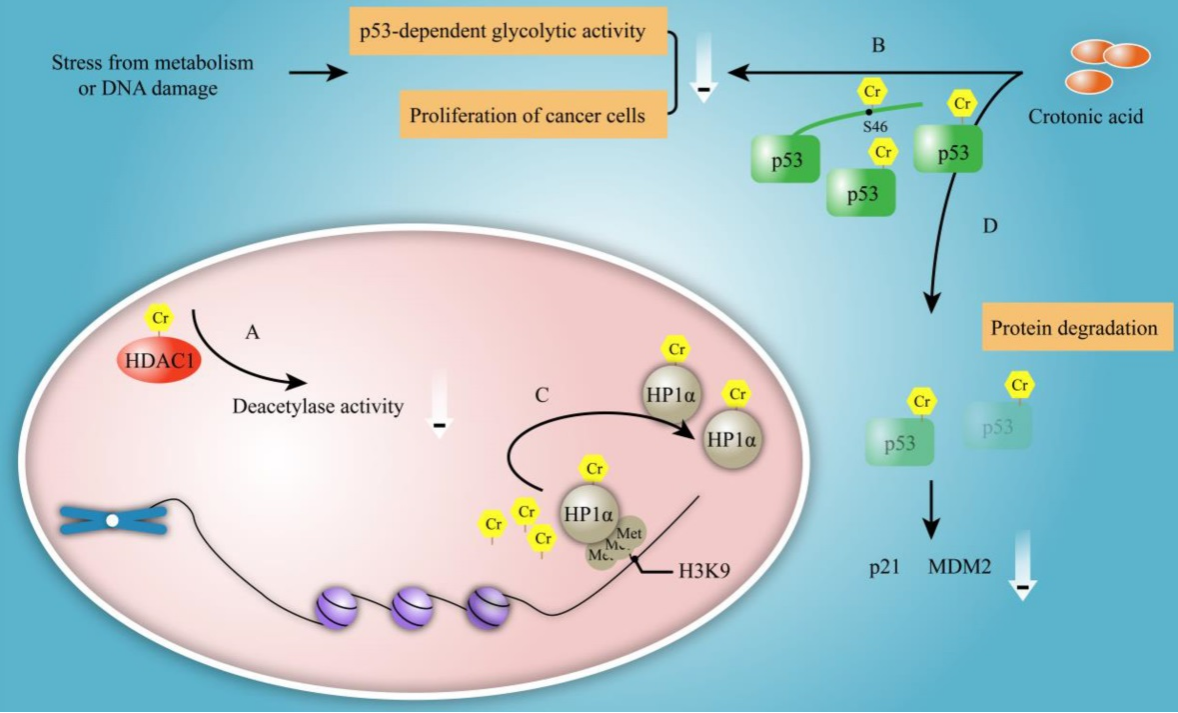
** The functional of non-histone crotonylation. A.** The crotonylation of HDAC1 can be enhanced by NaCr and crotonylated HDAC1 reduces its deacetylase activity.** B.** p53 is crotonylated at its serine 46 after CA treatment that leads to the inhibition of p53 activity in a dose dependent fashion. The crotonylation lowered activity of p53 enhances p53-dependent glycolytic activity and proliferation of cancer cells in response to stress from metabolism or DNA damage. **C.** Crotonylation of HP1α is shown to alter its localization from heterochromatin to nuclear plasm and dramatically reduces its binding of trimethylated H3 at K9 position, which results in variation of localization *in vitro*. D. CA treatment induces p53 crotonylation with the reduction of the protein level of p53 and its downstream protein p21 and MDM2.

The function of crotonylation on non-histone protein is paid more and more attention except for its roles on histone. Hundreds of crotonylated proteins and lysine residues have been identified using specific antibody enrichment followed by high-resolution mass spectrometry analysis. Bioinformatics analysis revealed that crotonylated proteins were particularly enriched for nuclear proteins involved in several physiological processes 38. The crotonylation of HDAC1 was enhanced by NaCr and the crotonylated HDAC1 exhibited reduction in its deacetylase activity compared with unmodified HDAC1 (Figure 7A). Also, p53 was found to be crotonylated at serine 46 after crotonic acid treatment that led to the inhibition of p53 activity in a dose-dependent fashion 10. The crotonylation of p53 lowered the activity of p53 protein to enhance the p53-dependent glycolytic activity and the proliferation of cancer cells in response to stress from metabolism or DNA damage 10 (Figure 7B).

#### Protein localization

Crotonylation has been reported to affect the localization of proteins. HP1α (also named chromobox protein homolog 5, CBX5) belongs to the heterochromatin family and enriches in heterochromatin by binding to methylated histones 38. HP1α is primarily localized at nucleus and accumulates at bright dot-like heterochromatin regions in HeLa cells. The localization of HP1α was changed from the heterochromatin to the nuclear plasm after treatment with either TSA or NaCr for 72 hours 38. For mechanism study, the authors found that NaCr treatment resulted in crotonylation of ectopically expressed Flag-HP1α by western blotting analysis. Also, the results of pulldown experiment showed that crotonylated HP1α dramatically reduced its binding of trimethylated H3K9 residue* in vitro* 38. These data provided evidence that crotonylation might affect the localization of proteins. Researcher speculated that crotonylated HP1α might be resulted in the variation of localization by reducing its binding of trimethylated H3 at K9 residue* in vitro* 38 (Figure 7C).

#### Protein degradation

There are multiple pathways to break down protein. Lysosomal pathway is an approach for protein degradation entered cell *in vitro*; while for intracellular proteins, the way of degradation is ubiquitin proteasome pathway. Many researches have shown that protein acylation is closely related to proteasome-dependent protein degradation 51, 52. Similarity to acetylation, whether crotonylation is associated with protein degradation or not? Liao et al. reported that crotonic acid induced the crotonylation of p53 and thus affected its degradation 10 (Figure 7D). Yet, the degradation pathways that were involved in the regulation of p53 degradation have not been identified. Furthermore, whether there is any association between crotonylation, acetylation and ubiquitination in protein degradation is poor understand. In the proteolytic pathway that relies on ubiquitin proteasome, acetylated protein was protected from ubiquitination and proteasome degradation 53. One possible mechanism is that acetylation and ubiquitination may compete for same lysine sites. In proteasome-independent degradation pathways, acetylation can also influence the stability of protein. Due to the similarity of crotonylation and acetylation, it needs further exploration concerning the effect of crotonylation on protein degradation.

### DNA repair

The repair of DNA damage is a complex process that relies on particular pathways to remedy specific types of damage to DNA. Crotonylation also plays a key role in DNA repair. In 2020, Yu et al. had identified replication protein A 70 kDa DNA-binding subunit (RPA1) as one of the downstream substrates of CDYL 54. They found that CDYL negatively regulated Kcr of RPA1 at K88, K379 and K595 sites, which were involved in DNA damage 54. Under the treatment of CPT, CDYL participated in up-regulation of Kcr in RPA1, which enhanced the interaction of RPA1 with ssDNA and/or HR factors. Combined with the physiological processes in the body, the researchers found that Kcr of RPA1 was important to cell survival and anti-apoptotic response under DNA-damaging conditions 54. The data above suggested that non-histone as well as histone crotonylation exhibited a key role in the process of DNA repair.

Crotonylation is an important epigenetic modification form identified as a novel evolutionarily conserved histone PTM. Both histone and non-histone proteins were found to be crotonylated. Crotonylation is involved in various biological pathways that regulate diverse cellular functions ranging from gene expression to DNA damage. It is interesting to determine how their functions are mechanistically regulated by crotonylation in future.

## Protein Crotonylation Associated Diseases

Kcr is a newly discovered post-translational modification, hence the associations between crotonylation and diseases are not fully clarified. More studies are needed to investigate the role of crotonylation in different diseases, which may provide new target for clinical therapy. The protein crotonylation associated diseases were summarized in Table 2.

### Depression

**Figure 8:**
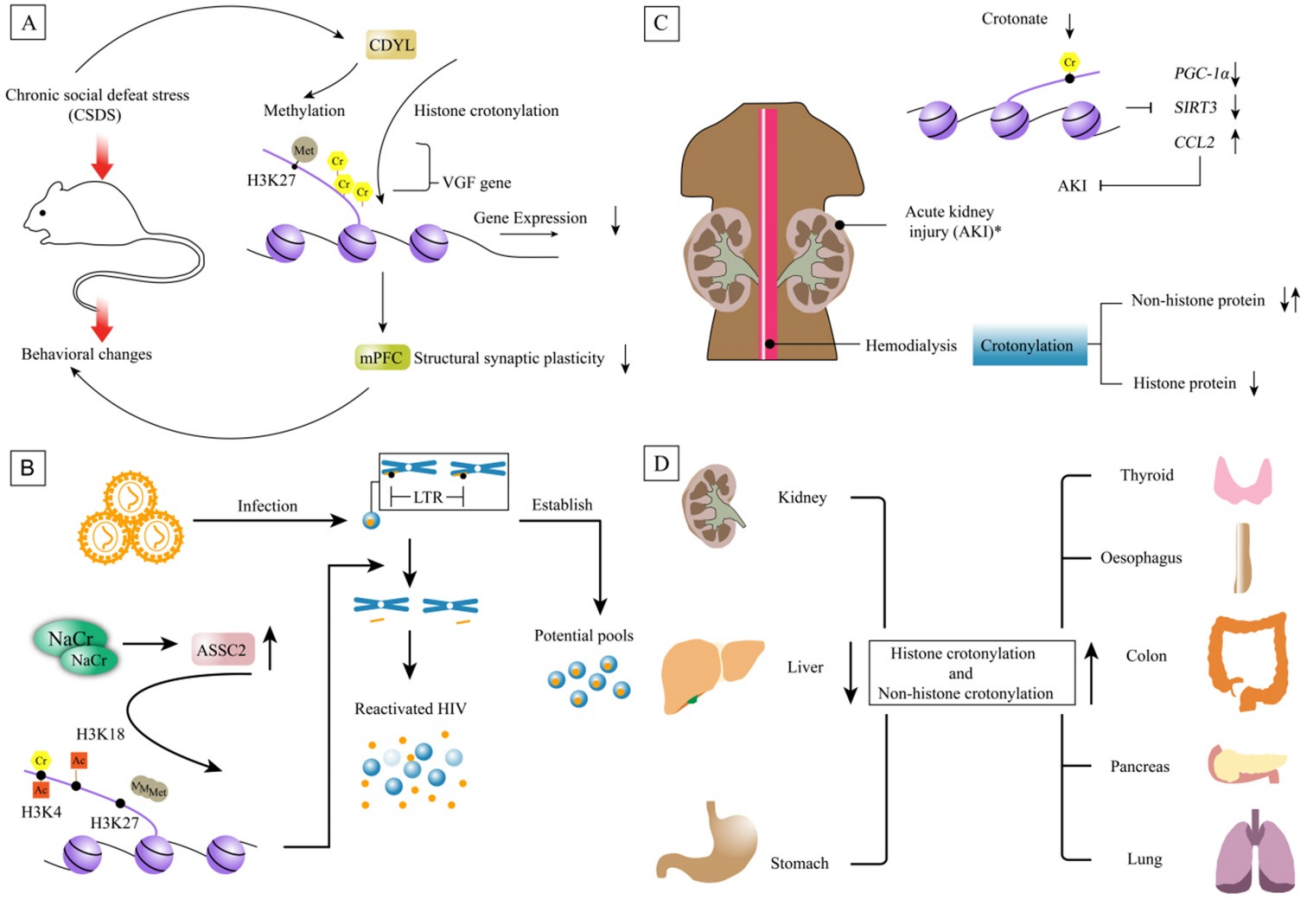
** Non-histone and histone crotonylation associated diseases. A.** The behavioral changes of rodents are caused by chronic social defeat stress. Because CDYL can make dual effect on histone crotonylation and H3K27 trimethylation on the VGF promoter. One of mechanism is that CDYL inhibits structural synaptic plasticity mainly by transcriptional repression of neuropeptide *VGF*. CDYL-VGF axis inhibits the structural synaptic plasticity of medial prefrontal cortex (mPFC), eventually leading to behavioral changes in susceptible individuals. **B.** NaCr increases the expression of ASSC2, thereby enhancing the expression of histone H3K4 crotonylation, H3K4 and H3K18 acetylation, and reducing H3K27 trimethylation. ACSS2-driven crotonylation of HIV LTR histones reshapes histone and reactivates HIV from the incubation period. ACSS2-driven crotonylation of histone where HIV LTR combine with DNA reshapes histone and reactivates HIV from the incubation period. **C.** Crotonate can protect experimental mice from AKI by preventing the decline of renal function, increase expression of renal *PGC-1α* and *SIRT3*, and decrease of *CCL2* though histone crotonylation. Note: this experiment was confirmed in mice. Proteome of crotonylation in maintenance hemodialysis patients (MHP) find that histone crotonylation is decreased in MHP. **D.** The whole level of crotonylation is down-regulated in liver, stomach and kidney cancers. However, it is upregulated in thyroid, oesophagus, colon, pancreas and lung carcinomas.

Depression is a psychological disease and the pathogenesis of depression is perplexing, but it is currently believed that depression is mainly determined by related genes and environmental factors. Also, PTM was shown to involve in this process, including acetylation, methylation, phosphorylation, and so on. For example, H3K14ac levels in hippocampus of male C57/Bl6J mice showed a significant increase after ten continuous days of social defeat stress 55. Another study revealed that HDAC was altered in the mood disorder patients. The expression of HDAC2, 3, 4 and 5 mRNA were increased in a depressive state in major depressive disorder patients. The expression of HDAC4 mRNA was increased only in a depressive state, and the expression of HDAC6, 7 and 8 were decreased in both depressive and remissive states in bipolar disorder patients, indicating that the altered expression of HDACs was associated with the pathophysiology of mood disorders 56. Interestingly, histone Kcr was reduced in the stress-susceptible rodents exposed in the medial prefrontal cortex concurrent with selective upregulation of CDYL 18. Mechanistically, they found that CDYL inhibited structural synaptic plasticity mainly by transcriptional repression of neuropeptide VGF nerve growth factor inducible through its dual effect on histone crotonylation and H3K27 trimethylation on the VGF promoter 18. CDYL-VGF axis inhibited the structural synaptic plasticity of medial prefrontal cortex (mPFC), eventually leading to behavioral changes in susceptible individuals (Figure 8A).

### HIV latency

Human immunodeficiency virus (HIV) is a retrovirus which causes a multisystemic disease called acquired immunodeficiency syndrome (AIDS). HIV are divided into two types: HIV-1 and HIV-2, of which the former is more pathogenic. People who get infection has a long incubation period and its long latency is a main reason for its incurability. Researchers have found that the epigenetic regulation of histone played a key role in the process of HIV latency. NaCr is a strong latent reversal agent (LRAs) to increase the expression of acyl-CoA synthetase short-chain family member 2 (ASSC2) *in vitro*, thereby enhancing histone H3K4 crotonylation, H3K4 and H3K18 acetylation, and reducing H3K27 trimethylation 19. ACSS2-driven crotonylation of histone where HIV long terminal repeat (LTR) combined with DNA reshaped histone and reactivated HIV from the incubation period 19. A similar phenomenon occurred in CD4^+^ T cells, and histone crotonylation combined with other LRAs interfered with HIV latency. In the rhesus monkey-AIDS animal model infected with simian immunodeficiency virus (SIV), the expression of ACSS2 was highly induced in the intestine during acute primary SIV infection* in vivo*. Entering the chronic stage, the expression of ACSS2 decreased 19 (Figure 8B). These results suggested a potential role of histone decrotonylation in the establishment of HIV latency.

### Kidney disease

Various epigenetic mechanisms are involved in different kidney diseases, such as acute kidney injury (AKI), chronic kidney disease (CKD) and AKI-CKD conversion 57. Histone crotylation is regulated by the concentration of crotonic acid and crotyl coenzyme A, and the crotonate has been applied to the folate-induced AKI model. By increasing histone crotonylation, crotonate reduced the inflammatory response and increased kidney function 16. Nevertheless, the mechanism regulated by crotonylation in kidney disease is still not clear and need to further explore.

#### Acute kidney injury (AKI)

Acute kidney injury (AKI) refers to sudden (within 1-7d) and sustained (>24 h) declines in renal function, manifested by azotemia, water electrolyte and acid-base imbalance, and systemic symptoms. Post-translational histone modifications modulate gene expression under the circumstance of AKI. Recently, Olga et al. observed that histone H3k9cr was increased in folic-acid-induced AKI tissue and disclosed that inflammatory factors may participate in regulation of histone crotonylation in kidney tubular cells 16. The results of ChIP-seq revealed enrichment of histone crotonylation at the genes encoding the mitochondrial biogenesis regulator *PGC-1α* and the decrotonylase *SIRT3* in both TWEAK-stimulated tubular cells and in AKI kidney tissue 16. Following these results, researchers evaluated the effect of variation in histone crotonylation on AKI. Crotonate heightened expression of *PGC-1α* and *SIRT3* and limited the expression of *C-C motif ligand 2* (*CCL2*) in healthy kidneys and renal tubular cell. Consistent with these results, crotonate protected experimental mice from AKI by preventing the decrease of renal *PGC-1α* and *SIRT3* and increase of *CCL2* 16 (Figure 8C). Cell stress and crotonate availability increased histone crotonylation* in vivo* and increasing histone crotonylation might have a protective effect from AKI.

#### IgA nephropathy

Immunoglobulin A nephropathy (IgAN) is one of the most common glomerular disease, characterized by IgA deposition, with or without the deposition of other immunoglobulins in the mesangial region. A proteomics analysis of crotonylation between healthy controls and IgAN patients was performed. By analyzing results in a bioinformatics manner, researchers focused on the characteristics of the crotonylayed proteins. Also, the functions of the differential crotonylayed proteins and the characteristics of the crotonylation sites in the amino acid sequence of proteins were analyzed. Integrated analysis revealed that crotonylated proteins were mainly involved in the humoral immune response in patients, especially in antigen processing and presentation 17.

#### Hemodialysis

Hemodialysis (HD) is one of the renal replacement treatments for patients with acute and chronic renal failure. Liquid chromatography tandem mass spectrometry (LC-MS/MS) coupled with highly sensitive immune-affinity purification was used to comparatively evaluate the crotonylation proteome of normal controls and maintenance hemodialysis patients (Figure 8C). There were 96 increased and 253 decreased crotonylated proteins have been confirmed, the decrease level of crotonylation in histones was disclosed in patients with kidney failure undergoing maintenance hemodialysis 58. With KEGG analysis, upregulated crotonylated proteins were found to involve in different physiological process of HD, such as complement and coagulation cascades, cardiac muscle contraction, and hematopoietic cell lineage 58. More researches are needed to disclose the relationships between crotonylation and the physiological and pathological processes in HD patients, which might provide a possible therapeutic target against HD complications and hopefully improve the quality of life of HD patients.

### Hypertrophic cardiomyopathy

Hypertrophic cardiomyopathy (HCM) is the most common genetic cardiovascular disease characterized by unexplained non dilated left ventricular hypertrophy (LVH). Recently, downregulation of short-chain enoyl-CoA hydratase (ECHS1) was showed in human hearts with hypertrophic cardiomyopathy. ECHS1 was reported to mediate histone crotonylation and contributed to cardiac homeostasis 59. They found that downregulation of ECHS1 increased the level of histone crotonylation of H3K18 and H2BK12, and enhanced the expression of genes related to myocardial hypertrophy, such as *natriuretic peptides B (Nppb*), ultimately leading to the development of HCM 59. Hopefully, it provides a potential target for treatment of HCM.

### Cancer

Cancer is a major public health problem and owns high leading cause of death worldwide^60^. Epigenetics is one of the major focus and extensive research on histone methylation, acetylation, and phosphorylation has been performed. Recently, a quantitative proteomics study characterized the p300-regulated lysine crotonylome and showed that p300-targeted Kcr substrates the potentially linked to cancer 61. These suggest that crotonylation may act as a carcinogenic factor to promote tumor progress. In the EDRN database, 4.5% (20 out of 443) of tumor biomarkers have been crotonylated and 32 crotonylated proteins are associated with tumor genes 61. The crotonylated proteins associated tumor genes are important for tumorigenesis and tumor development. In adrenal aldosterone producing adenoma, the crotonylated protein associated gene is *ATPase Na^+^/K^+^ transporting subunit alpha 1* (*ATP1A1*). In Spitzoid tumor, the genes are *lamin A/C* (*LMNA*) and* lamin B2* (*LMNB2*). In anaplastic large cell lymphoma, the genes related protein crotonylation are *moesin* (*MSN*), *myosin heavy chain 9* (*MYH9*) and *myosin heavy chain 10* (*MYH10*) 61. The role of crotonylation on these proteins associated with tumor genes needs to be further explored^61^. Also, p300 was shown to mediate the expression of HnRNPA1 by lysine crotonylation to further promote the proliferation, invasion and migration of HeLa cells 62. Furthermore, the level of crotonylation in several tumors were detected and found that the level of crotonylation was down-regulated in liver cancer, gastric cancer and renal cancer, while it was upregulated in thyroid, oesophagus, colon, pancreas and lung carcinoma 20 (Figure 8D). In hepatocellular carcinoma, the expression of Kcr was correlated with cancer staging 20. In addition to histone crotonylation, numerous non-histone proteins are involved in the process of tumorigenesis. 2 696 crotonylation sites were identified in total on 1024 proteins of the human lung adenocarcinoma H1299 cell line 25. Enrichment analysis of biological processes indicated that these crotonylated proteins are enriched in a variety of biological processes including transport, nucleobase-containing compound metabolic process and heterocycle metabolic process of lung adenocarcinoma cell line 25. These data suggested that non-histone crotonylation might be play a critical role in tumorigenesis.

## Conclusion and Perspectives

Kcr is one of the non-acetyl lysine acylation modifications, which caused more and more concern. This article generalizes the related researches on crotonylation over decades by discussing the research processes of crotonylation and its involved physiological and pathological function. Particularly, we summarized histones and non-histones protein crotonylation in combination with specific modification sites, which shows that protein crotonylation is widely distributed in cells and it has significant roles in countless physiological processes. Lysine histone and non-histone proteins crotonylation is involved in many physiological processes from gene expression to protein stability. Also, the histone and non-histone proteins crotonylation associated diseases were discussed deeply here. It will be interesting to determine how their functions are mechanistically regulated by crotonylation in the near future.

Studies have shown that acetylation and crotonylation coexist in TSS or other regulatory elements of certain genes. This phenomenon suggests that there are intricate correlations between histone crotonylation and other types of histone modifications in the regulation of gene expression* in vivo*. The mechanism of crotonylation and its dynamic relationship with other acylation needs further exploration.

Until now, drugs that target histone crotonylation are still not available. Countless reasons contribute to its perplexity. Due to complex biochemical reactions of ester drugs metabolism in body, the lack of specific crotonylated enzyme is a main challenge, which limits further investigation of drugs targeting histone crotonylation. Despite the obstacles, studies on non-histone crotonylation made breakthroughs with the developments in high‐resolution LC‐MS/MS approaches. In 2017, Z Ju et al. designed the new coding schemes to predict protein crotonylation sites through databases 63, 64. In 2018, J Bos et al. designed a chemical probe for crotonylation on endogenous protein as a supplement to specific antibodies to detect crotonylation modifications 65. The combination of traditional biochemical technology with bioinformatics method will be benefit to investigate the function of protein crotonylation on many cellular processes mechanistically. While understanding the roles of crotonylation in physiological processes and in the development of diseases, it will guide the development of drugs that target of protein crotonylation and provide us a new theoretical basis for clinical diseases therapy.

## Figures and Tables

**Table 1:** The significance of histone crotonylation sites reported in gene expression

Histone crotonylation Sites	Activation/Inactivation	Description	References
**H2B**			
K12	activation	CDYL down-regulated gene expression through decreasing H2BK12cr on the promoter of genes such as *BDNF*, *NEUROD1*, *SCG10*, and *MYT1*. Downregulation of ECHS1 increased the level of histone crotonylation of H2BK12 and enhanced the expression of genes related to myocardial hypertrophy.	22,59
**H3**			
K9	inactivation	Increased H3K9cr by crotonic acid suppressed the expression of growth-related genes.	33
K18	activation	Downregulation of ECHS1 increased the level of histone crotonylation of H3K18 and enhanced the expression of genes related to myocardial hypertrophy.	59
K27	activation	H3K27cr located in the regulatory elements of genes, and participated in the expression of genes, which are important for the proper differentiation of round spermatids into final sperm.	67

**Table 2:** The diseases associated with crotonylation

Diseases	Protein crotonylation	Description	References
Depression	Histone	CDYL may inhibit structural synaptic plasticity mainly by transcriptional repression of neuropeptide *VGF*. CDYL-mediated histone crotonylation played a critical role in regulating stress-induced depression	18
HIV latency	H3K4	Histone H3K4 crotonylation by ACSS2 inducted reprogramed histone tails at the HIV LTR and suppressed ACSS2 dampened reactivation of latent HIV.	19
**Kidney disease**			
Acute kidney injury (AKI)	Histone	Crotonate protected experimental mice from AKI by preventing the decline of renal function, increased expression of renal *PGC-1α* and *SIRT3*, and decreased of *CCL2* though histone crotonylation.	16
IgA nephropathy	353 crotonylated proteins	Bioinformatics analysis identified 353 crotonylated proteins. Genomes and functional enrichment analyses suggested significant enrichment of crotonylated proteins displaying important relationships with IgA nephropathy.	17
Hemodialysis	347 crotonylated proteins	Proteome of crotonylation was confirmed to evaluate the role of lysine crotonylation in maintenance hemodialysis patients (MHP) and found that lysine crotonylation played important regulatory roles in pathophysiological processes in MHP.	58
Hypertrophic cardiomyopathy	H3K18; H2BK12	Downregulation of ECHS1 increased the level of histone crotonylation of H3K18 and H2BK12, and enhanced the expression of genes related to myocardial hypertrophy, such as natriuretic peptides B (*Nppb*), ultimately leading to the development of HCM.	59
Cancer	Numerous non-histone proteins	Quantitative proteomics study showed that p300-targeted Kcr substrates owned the potentially linked to cancer. Also, numerous non-histone proteins were shown to involve in the process of tumorigenesis by LC-MS/MS.	20,62
